# Time-Dependent Response of Human Deciduous Tooth-Derived Dental Pulp Cells Treated with TheraCal LC: Functional Analysis of Gene Interactions Compared to MTA

**DOI:** 10.3390/jcm9020531

**Published:** 2020-02-15

**Authors:** Ok Hyung Nam, Jae-Hwan Kim, Sung Chul Choi, Young Kim

**Affiliations:** 1 Department of Pediatric Dentistry, School of Dentistry, Kyung Hee University, Seoul 02447, Korea; pedokhyung@gmail.com (O.H.N.); pedochoi@khu.ac.kr (S.C.C.); 2 Department of Pediatric Dentistry, School of Dentistry, Chonnam National University, Gwangju 61186, Korea; jhbcss@hanmail.net; 3 Department of Oral Pathology, School of Dentistry, Chonnam National University, Gwangju 61186, Korea

**Keywords:** gene expression, mineral trioxide aggregate, mRNA-sequencing, TheraCal LC, vital pulp therapy

## Abstract

Pulp capping material should facilitate hard tissue regeneration on the injured pulp tissue. TheraCal LC (TC) was recently developed. Although TC has shown reliable clinical outcomes after direct pulp capping, there are still remaining concerns regarding its detrimental effect on pulp cells. Therefore, this study aimed to identify the gene expression of human deciduous tooth-derived dental pulp cells exposed to TC compared to mineral trioxide aggregate (MTA). The cells were cultured and exposed to TC and MTA for 24 and 72 h. Next, total RNA was isolated. QuantSeq 3′ mRNA-sequencing was used to examine differentially expressed genes (DEGs) in exposed to TC and MTA. Functional analysis of DEGs was performed using bioinformatics analysis. In gene ontology (GO) functional enrichment analysis, cells in TC for 24 h presented significantly enriched immune response (*p* < 0.001) and inflammatory response (*p* < 0.01) compared to MTA. TC showed enriched positive regulation of cell migration at 72 h (*p* < 0.001). In Kyoto Encyclopedia of Genes and Genomes (KEGG) analysis, neuroactive ligand–receptor interaction (*p* = 1.19 × 10^−7^) and calcium signaling pathway (*p* = 2.96 × 10^−5^) were confirmed in the shared DEGs in TC. In conclusion, DEGs in TC may be involved in pathways associated with osteoclastogenesis and osteoclastic differentiation.

## 1. Introduction

Vital pulp therapy is a dental terminology that aims to preserve pulp tissue damaged by dental caries or traumatic dental injuries [1]. Among possible modalities included in vital pulp therapy, direct pulp capping (DPC) is particularly indicated when the pulp tissue is exposed. DPC is a procedure that applies a pulp capping material onto a pulp exposure site for hard tissue regeneration (reparative dentin formation) [2].

In the case of pulpal exposures, dental pulp stem cells (DPSCs) in the pulpal tissue can proliferate and differentiate into odontoblasts, facilitating hard tissue regeneration [3]. Although DPSCs have potentials to form a hard tissue barrier, pulp capping materials also promote hard tissue regeneration and prevent bacterial invasion [4]. Thus, pulp capping materials should be considered on the basis of biocompatibility, antibacterial, and anti-inflammatory properties, and bioactivity that promotes DPSC activity and pulp tissue healing [5,6,7,8].

Calcium hydroxide has traditionally been used as a pulp capping material for DPC. However, the reparative dentin formed by calcium hydroxide is porous and susceptible to bacterial invasion [9]. Recently, mineral trioxide aggregate (MTA) has served as a good pulp capping material. MTA is a calcium silicate cement with good antibacterial properties, sealing ability, and biocompatibility [10]. Previous studies showed that MTA can stimulate DPSC differentiation [11]. However, MTA requires several hours for setting and can cause tooth discoloration [12,13]. Several pulp capping materials have been developed to overcome MTA’s drawbacks. TheraCal LC (TC) is a resin-modified MTA-based material that can easily be set using light curing. TC has good bioactivity with reduced incidence of tooth discoloration compared to MTA [14,15]. Since TC contains resin monomers, there are still concerns regarding its detrimental effect on DPSCs [16]. A previous study on the cytocompatibility of various pulp capping materials demonstrated that TC showed the lowest cytocompatibility compared to MTA and Biodentine [17].

Recent advances in RNA sequencing technology can provide more accurate measurement of transcript levels. RNA sequencing is extremely sensitive and accurate in detecting and quantifying gene expression levels compared to other methods [18]. Thus, exploring time-dependent changes in gene expression profiles can more precisely identify the dynamic response of pulp cells exposed to pulp capping materials.

While the effect of various pulp capping materials on DPSCs have been extensively identified, the pulp cell response from primary teeth is still unclear. Considering the abundance of undifferentiated mesenchymal stem cells present in the pulp tissue of primary teeth [19], the effect of pulp capping materials on human deciduous tooth-derived dental pulp cells should be investigated. Therefore, the purpose of this study was to identify the genetic response of human deciduous tooth-derived dental pulp cells exposed to TC and MTA and to predict the function of gene–gene interactions with time-dependent manner.

## 2. Materials and Methods

### 2.1. Cell Isolation and Culture

Physiologically exfoliated human deciduous teeth from healthy children were collected from the Department of Pediatric Dentistry, Chonnam National University Dental Hospital, under approval of the Ethics Committee of Chonnam National University Dental Hospital, Gwangju, Republic of Korea (CNUDH-2013-002). Pulp tissue was removed from the teeth using a barbed broach and placed in Dulbecco’s phosphate-buffered saline (PBS) solution (WELGENE, Gyeongsan-si, Geyongsangbuk-do, Korea) to isolate and culture dental pulp cells. The pulp tissue was washed three times with PBS and cut into small fragments before resuspending in alpha-minimum essential medium (α-MEM, Gibco Invitrogen, Grand Island, NY, USA) supplemented with 10% fetal bovine serum (FBS), 100 U/mL penicillin, and 100 mg/mL streptomycin. Cells were seeded into 6-well plates and incubated at 37 °C in a humidified atmosphere containing 5% carbon dioxide (CO2). After reaching confluency, the cells were passaged using trypsin. The cells at passage 3 to 5 were used for the experiments.

### 2.2. Cell Viability Test

Cell viability was determined using the water-soluble tetrazolium salt (WST) based assay (EZ-CYTOX; Daeil Lab, Seoul, Korea). The cells were seeded onto 96-well plates with α-MEM with 10% FBS at 2 × 104 cells/well and were exposed to experimental materials for either 24 or 72 h. Various concentrations of the experimental materials were tested, including dilution ratios of 1/100, 1/50, 1/5, and 1. After 24 h of incubation, 10 µL tetrazolium salt reagent (EZ-CYTOX) was added to each well and incubated for another 4 h at 37 °C. The optical density (OD) of each well was determined at 450 nm on a multi-well plate reader (Multiskan GO, Thermo scientific, Waltham, IL, USA). Cell viability was measured as a ratio of optical density from experimental material to control (no treatment). For statistical analysis, the Kruskal–Wallis test was performed and Mann–Whitney test was performed as post hoc analysis. *p*-values <0.05 indicated statistical significance.

### 2.3. Material Preparation

The materials used in this study were MTA (ProRoot MTA; Dentsply, Tulsa Dental, Tulsa, OK, USA) and TC (Bisco Inc., Schaumburg, IL, USA). MTA and TC were prepared according to the manufacturer’s instructions under aseptic conditions. Next, each material was molded into a polyethylene tube (5 mm diameter, 3 mm height). TC was light cured for 120 s for sufficient curing level and maintained for 24 h at 37 °C in a humidified atmosphere containing 5% CO_2_. MTA was allowed to set for the same condition with TC. The samples were sterilized using ultraviolet radiation. α-MEM (50 ml) supplemented with 1% penicillin and streptomycin was added to the molded materials for infusion. Before use, undiluted, 1/5, 1/10, 1/50, and 1/100 dilutions of the materials were prepared and filtered using a 0.2 μm syringe filter.

### 2.4. RNA Isolation and Library Preparation for Next Generation Sequencing

Total RNA was isolated from the prepared cells using TRIzol reagent (Invitrogen, Carlsbad, CA, USA). The integrity of the total isolated RNA was assessed using an Agilent 2100 bioanalyzer with an RNA 6000 Nano LapChip kit (Agilent Technologies, Amstelveen, The Netherlands). The amount of RNA was quantified using an ND-2000 Spectrophotometer (Thermo Inc., Wilmington, DE, USA). Control and test RNA libraries were constructed using a QuantSeq 3′ mRNA-Seq Library Prep Kit (Lexogen Inc., Vienna, Austria) according to the manufacturer’s instructions. Briefly, 500 ng of total RNA for each sample was used. An oligo-dT primer containing an Illumina-compatible sequence at the 5′ end was hybridized to the RN before performing cDNA synthesis using reverse transcription. Second strand synthesis was initiated using random primers containing an Illumina-compatible linker sequence at the 5′ end. The double stranded library was purified using magnetic beads to remove all reaction components before amplifying to add the complete adapter sequences required for cluster generation. The finished library was purified from PCR components. High-throughput sequencing was performed on single-read 75 bp fragments using a NextSeq 500 (Illumina Inc., San Diego, CA, USA).

### 2.5. Data Processing to Identify DEGs and GO Functional Enrichment Analysis

QuantSeq 3′ mRNA-Seq reads were aligned using Bowtie2 [20]. Bowtie2 indices were either generated from the genome assembly sequences or representative transcript sequences that aligned to the genome and transcriptome. The alignment file was used to assemble transcripts, estimate transcript abundance, and detect differential gene expression. DEGs were determined based on the counts from unique and multiple alignments using coverage in Bedtools [21]. Read count data were processed using the quantile normalization method in EdgeR, a Bioconductor package in R (R development Core Team, 2016) [22]. The cutoff threshold for DEGs was set at 2-fold change.

Gene classification was based on the Database for Annotation, Visualization and Integrated Discovery (DAVID) bioinformatics software 6.8 (http://david.abcc.ncifcrf.gov/) and Medline databases (http://www.ncbi.nlm.nih.gov/). Using DAVID tools, enriched GO function including BPs, MFs, and CCs were identified based on *p*-values of <0.05. All mRNA-seq data were deposited in GEO (No. GSE139497).

### 2.6. GSEA and PPI Network Construction

GSEA was performed on selected data using the online GSEA 4.0.0. program (http://software.broadinstitute.org/gsea/index.jsp) [23]. To assess KEGG pathways and pathway genes, we used gene sets with FDR set at *q*-value <0.05 [24]. For analyzing the molecular interactions, the STRING was employed to construct a PPI network. The minimum required interaction score was set at the highest confidence interval (>0.900), and the inflation parameter was set as 3 for MCL clustering.

## 3. Results

### 3.1. Cell Viability Test

**Figure 1:**
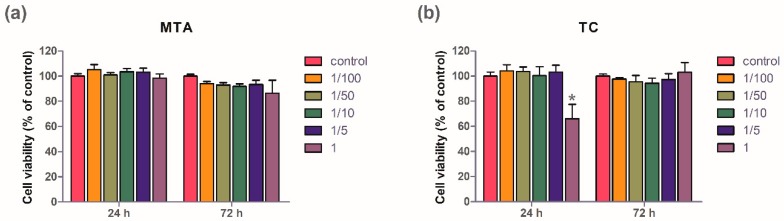
Water-soluble tetrazolium salt (WST) assay of mineral trioxide (MTA) and TheraCal LC (TC). The cell viability under different conditions was evaluated using the WST assay. The results are shown as the ratio of optical density at 450 nm of culture medium from cells exposed to pulp capping materials compared to control cell culture medium. (**a**) Cell viability of MTA under different concentrations. (**b**) Cell viability of TC under different concentrations. The cell viability significantly decreased when the cells were exposed to TC at a concentration of 100% for 24 h. * *p* < 0.05.

Figure 1 shows cell viabilities exposed to MTA and TC under different conditions. There were no significant differences in the cell viabilities exposed to MTA under different conditions for any duration. However, the cell viability was significantly decreased when the cells were exposed to TC at a concentration of 100% for 24 h (*p* < 0.05).

### 3.2. Overview of Differentially Expressed Genes (DEGs) in TC

**Figure 2:**
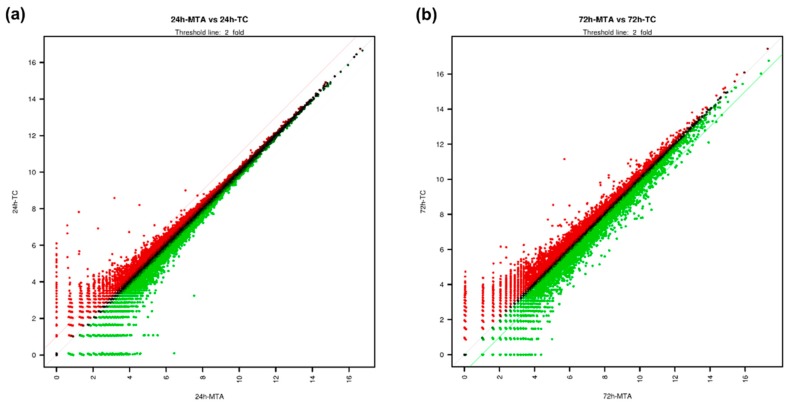
Scatter plot analysis. In each plot, the central line passing through the origin indicates no difference in expression between the cells stored for different materials. Values above the red line and below the green line indicate a more than 2-fold difference. (**a**) Scatter plot analysis of MTA and TC for 24 h. (**b**) Scatter plot analysis of MTA and TC for 72 h.

**Figure 3:**
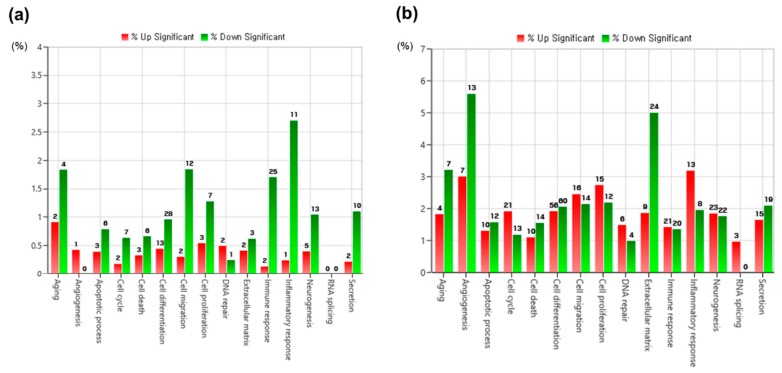
Overviews of differentially expressed genes (DEGs) in TC compared to MTA. (**a**) Gene category chart in the group exposed to TC for 24 h. (**b**) Gene category chart in the group exposed to TC for 72 h. The distributions of upregulated and downregulated DEGs according to exposure time are presented. The number on top of each bar refers to the number of identified genes with the corresponded gene category. Only DEGs with more than a 2-fold change are shown in the gene category charts.

Here, 4711 genes with at least a 2-fold difference were identified out of a total of 25,737 genes examined. Scatter plot analysis results showed different DEG distributions between TC and MTA. The expression of most genes showed a less than 2-fold difference between TC and MTA (Figure 2). Gene category charts showed that the DEG distribution varied according to exposure time (Figure 3). In the charts, the number of upregulated and downregulated DEGs in TC increased over time compared to MTA.

### 3.3. Gene Ontology (GO) Functional Enrichment Analysis

**Figure 4:**
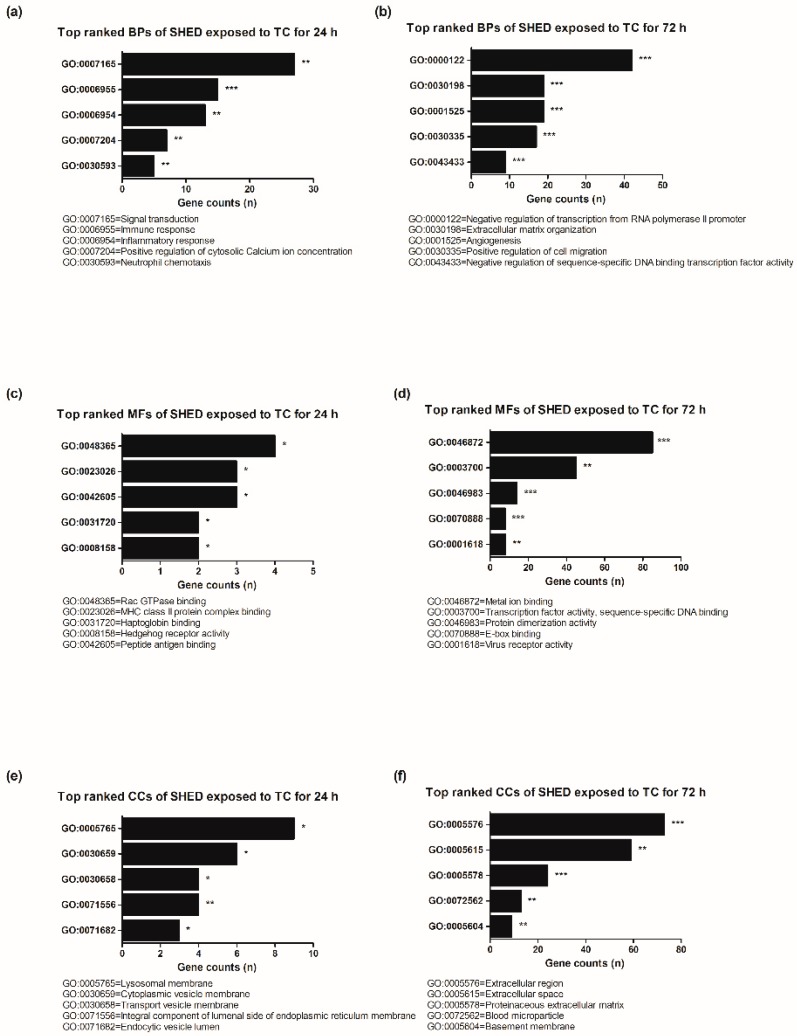
Database for Annotation, Visualization and Integrated Discovery (DAVID) functional gene ontology analysis of protein enrichment in TC compared to MTA. (**a**,**b**) Biological process of the cells exposed to TC. (**c**,**d**) Molecular function of the cells exposed to TC. (**e**,**f**) Cellular components exposed to TC. BP = biological process, MF = molecular function, CC = cellular component, * *p* < 0.05, ** *p* < 0.01, *** *p* < 0.001.

The cells exposed to TC for 24 h were significantly enriched in biological processes including immune response, signal transduction, inflammatory response, positive regulation of cytosolic calcium ion concentration, and neutrophil chemotaxis compared to MTA for 24 h (Figure 4a). In the cells exposed to TC for 72 h, extracellular matrix organization, negative regulation of transcription from RNA polymerase II promoter, positive regulation of cell migration, angiogenesis, and negative regulation of sequence-specific DNA binding transcription factor activity were enriched (Figure 4b).

Regarding significantly enriched molecular functions, Rac GTPase binding, MHC class II protein complex binding, hedgehog receptor activity, haptoglobin binding, and peptide antigen binding were overrepresented in the cells exposed to TC for 24 h compared to MTA for 24 h (Figure 4c). In the cells exposed to TC for 72 h, E-box binding, protein dimerization activity, metal ion binding, transcription factor activit -sequence-specific DNA binding, and virus receptor activity were overrepresented (Figure 4d). Figure 4e,f show significantly enriched cellular components in TC compared to MTA for either 24 h or 72 h.

### 3.4. Kyoto Encyclopedia of Genes and Genomes (KEGG) Pathway Enrichment Analysis

**Figure 5:**
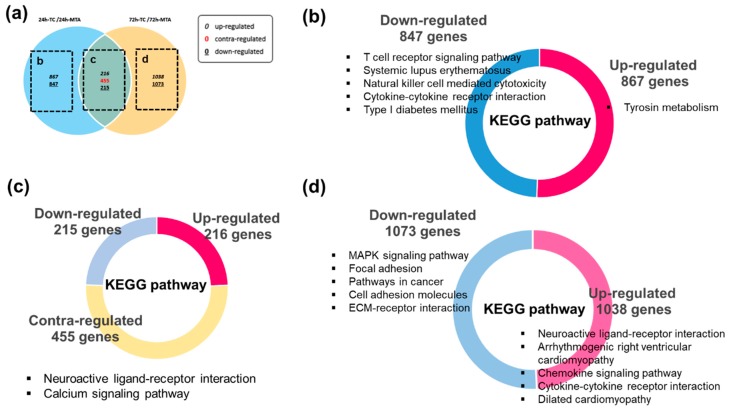
Kyoto Encyclopedia of Genes and Genomes (KEGG) pathways significantly enriched between TC and MTA. (**a**) Venn diagram analysis of the DEGs in TC compared to MTA. (**b**) KEGG pathways significantly enriched in TC for 24 h. (**c**) KEGG pathways significantly enriched in TC between 24 h and 72 h. (**d**) KEGG pathways significantly enriched in TC for 72 h. The identified KEGG pathways are presented according to gene expression regulation.

KEGG pathway enrichment analysis was performed to assess DEG functional annotations. Here, 33 significantly enriched KEGG pathways were identified based on the gene set enrichment analysis (GSEA). Figure 5 illustrates the top five KEGG pathways according to each section. Two enriched pathways were identified in the shared DEGs between TC 24 h and 72 h, including neuroactive ligand–receptor interaction and the calcium signaling pathway. Table 1 and Table 2 show the details of the identified KEGG pathway involved in TC in a time-dependent manner. The DEGs involved in KEGG pathway in TC for both 24 h and 72 h are summarized in Table 3, Table A1 and Table A2.

### 3.5. Gene Network Construction

**Figure 6:**
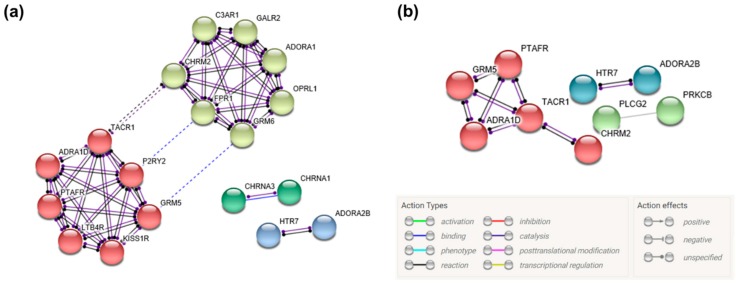
Gene network construction of DEGs involved in TC using Search Tool for the Retrieval of Interacting Genes (STRING). The minimum required interaction score was set as the highest confidence value (>0.900), and the inflation parameter was set to 3 for MCL clustering. (**a**) Gene networks of DEGs involved in neuroactive ligand–receptor interaction pathway. (**b**) Gene networks of DEGs involved in the calcium signaling pathway. Colored nodes represent query proteins and first shell of interactors.

The protein–protein interaction (PPI) network was constructed using the Search Tool for the Retrieval of Interacting Genes (STRING) to analyze the gene network. Among identified DEGs, we constructed only PPIs for DEGs involved in two KEGG pathways: The neuroactive ligand–receptor interaction and calcium signaling pathways (Figure 6). The PPI network of DEGs involved in the neuroactive ligand–receptor interaction pathway contained 22 nodes, 47 edges, and the average local clustering coefficient was 0.74. The PPI enrichment *p*-value was 1.0 × 10^−16^. The PPI network of DEGs involved in the calcium signaling pathway contained 14 nodes, 9 edges, and the average local clustering coefficient was 0.607. The PPI enrichment *p*-value was 0.00072.

## 4. Discussion

Previously, DPC was not generally recommended for primary teeth [25]. However, high DPC clinical success rates have been reported in primary teeth with advances in pulp capping materials [2,26,27]. The studies of DPC with MTA in primary teeth reported 93.8% to 100% DPC success rates with MTA [2,27]. A previous study compared clinical and radiographic success between MTA and TC in primary teeth [26]. It concluded that both materials had higher than 90% success rate, and the success rate was not significantly different. Even though TC showed a high clinical success rate on DPC in primary teeth, there still remain concerns about TC-induced cell toxicity. This is because TC contains resin monomers as the main component (45%), including bisphenol A-glycidyl methacrylate (Bis-GMA) and urethane dimethacrylate (UDMA) [28]. These resin monomers can exert toxicity in dental pulp cells [16].

Cell viabilities after TC and MTA exposure were measured in this study. The cell viability of MTA was highly maintained for 72 h regardless of MTA concentrations. Interestingly, the cell viability of TC with no dilution was significantly decreased compared to control. This finding corresponded with the previous studies which reported lower cell viability in TC-conditioned stem cells from human exfoliated deciduous teeth (SHED) compared to MTA-conditioned or Biodentine-conditioned SHED [8,29].

Regarding identified DEGs, scatter plot analysis revealed that most DEGs were shared in both MTA and TC. Gene category chart showed that the number of upregulated and downregulated DEGs in TC increased over time compared to MTA. These findings indicate that some biological behaviors of TC may differ by changes in gene expression over time.

In this study, GO functional enrichment analysis provided comprehensive information about interactions between TC and human deciduous tooth-derived dental pulp cells. The cells in TC for 24 h showed a significantly enriched immune response (*p* < 0.001) and inflammatory response (*p* < 0.01) in the biological processes compared to MTA. Two enriched biological responses here may be associated with repair process of injured pulp tissue. This is because immune responses associated with the inflammatory response are essential in bone regeneration and tissue repair [30,31]. Thus, pulp capping materials should induce pulp cell migration for hard tissue regeneration [8]. However, TC did not show positive gene expression of cell migration ability for 24 h in this study. Among molecular functions identified by GO functional enrichment analysis, the hedgehog pathway was significantly enriched in SHED in TC for 24 h (*p* < 0.05). The hedgehog signaling pathway is required during tooth development and odontogenesis [32,33]. Previous studies reported that the hedgehog signaling pathway can promote odontoblastic differentiation in human DPSCs [33,34].

GSEA was performed to predict potential DEG functions, and KEGG pathways were verified: Neuroactive ligand–receptor interaction and calcium signaling pathway. Regarding neuroactive ligand–receptor interaction, this pathway can affect several aspects of cell behaviors. This pathway regulates tooth morphogenesis during tooth development [35]. A previous animal study with Raptor/mTORC1 knockout mice revealed that the animals presented less dentinogenesis [36]. The study further performed RNA sequencing analysis to assess gene expression profiles. Mesenchymal stem cells from molar tooth dental papilla were used. The analysis showed that downregulation of differentiation and cell proliferation-related genes and neuroactive ligand–receptor interaction may be associated with this phenomenon. Moreover, a previous monocyte-driven gene expression study of Chinese women with different bone mass indices demonstrated that neuroactive ligand–receptor interaction was the key pathway involved in osteoclastogenesis. This pathway may have a close relationship with osteoporosis development [37].

Among identified DEGs involved in neuroactive ligand–receptor interaction, the potential effects of the DEGs were different. This could be attributed to the uncovered vast network of gene interactions due to coupling with many genes that are also associated with this pathway (See Figure 6). Complement C3a receptor 1 (C3AR1) is downregulated in TC for 24 h and reversed after 72 h, which was contrary to regulation by MTA in this study. Downregulation of C3AR1 can negatively affect hard tissue regeneration after TC application. C3AR1′s role is regarded as increasing DPSCs and fibroblast proliferation by mobilizing DPSCs and guiding fibroblast recruitment. This role is believed to be important in the early steps of pulp dentine regeneration [38].

On the other hand, adenosine A1 receptor (ADORA1), adenosine A2b receptor (ADORA2B), and purinergic receptor P2Y2 (P2RY2) upregulation was shown in TC in 24 h. The upregulation of these receptors was more pronounced in TC compared to MTA in 24 h. The upregulation is postulated to positively affect TC on hard tissue regeneration after TC application. The purinergic signaling pathway regulates proliferation and differentiation of various stem cells. Adenosine triphosphate and its hydrolysates act through purinergic receptors. P2Y2 is mainly regulated by ATP. A previous study showed that ATP-induced odontoblastic differentiation of human pulp cell may be enhanced by the combination of ADORAs and P2Y2 [39].

The calcium signaling pathway plays a critical role in osteoclast differentiation and function [40]. Regarding identified genes involved in the calcium signaling pathway, calcium/calmodulin dependent protein kinase IV (CAMK4) expression was more pronounced, and glutamate metabotropic receptor 5 (GRM5) was downregulated in TC for both exposure durations compared to MTA. This finding indicates that human deciduous tooth-derived dental pulp cells exposed to TC may be more susceptible to differentiate into osteoclast compared to MTA. Receptor activator of nuclear factor-κB (RANK)/RANK ligand (RANKL) is a central initiating signaling pathway for osteoclastogenesis. This RANK/RANKL pathway is primarily modulated by the calcium signaling pathway via Ca^2+^ dependent calcineurin/NFAT pathway [41,42]. Nuclear factor of activated T cells cytoplasmic 1 (NFATc1) is known as the master regulator of osteoclast differentiation, and it is activated by the calcium signaling pathway [43]. CAMK4 induces NFATc1 expression in the activation of osteoclast-specific genes [44]. Also, GRM5 is physically associated with calcineurin through intermediate linking of both proteins and calmodulin. Calmodulin is necessary for calcineurin activation [45]. However, phospholipase C gamma 2 (PLCG2) was upregulated with increasing time in both MTA and TC. PLCG2 inhibits RANKL-induced osteoclastogenesis [46].

In the significantly enriched KEGG pathways in TC for 24 h, the downregulated DEGs in TC compared to MTA were correlated with the T cell receptor signaling pathway and natural killer cell-mediated cytotoxicity. The immunomodulating capacity of biomaterials is beneficial for ensuing biocompatibility and performance [47]. This concept has been strengthened by previous studies about biomaterial immunomodulation. The previous studies reported that decreased NK cell activity by innate and adaptive immune cells was observed in response to biomaterials [48,49]. In addition, a previous study demonstrated that DPSC and DPSC differentiation protection is increased by increasing release of IFN-γ and decreasing cytotoxic function of natural killer cells. In addition, the increase in IFN-γ secretion affects increasing antigen-specific T cell functions and initiation of adaptive immunity [50].

In this study, downregulated DEGs in TC for 72 h compared to MTA were correlated with the mitogen-activated protein kinase (MAPK) signaling pathway. These findings show that TC can affect odontogenic differentiation via the MAPK signaling pathway. MAPKs are composed of three-enzyme families: Extracellular signal-regulated kinases (ERKs), c-Jun amino-terminal kinases (JNKs), and p38 MAPK [51]. MAPKs regulate fundamental responses of mammalian cells like growth, proliferation, differentiation, and apoptosis [52]. MAPK pathway activation through p38 phosphorylation acts as a molecular switch to modulate odontoblast secretion [53]. Thus, the MAPK signaling pathway, especially p38 MAPK, is essential to the transcriptional control of odontoblast secretion [54]. It was also reported that MTA induces and promotes odontogenic differentiation via the MAPK signaling pathway [4,55,56].

Within the limits of this study, the functions of TC-induced gene–gene interactions were related to the pathways and may be associated with osteoclastogenesis and osteoclastic differentiation. These findings support that TC should be applied with strong caution. Evidence indicated that some resin monomers incorporated in TC may not be polymerized during light curing, and they can be released and pose a detrimental effect on pulp cells [29]. Therefore, it is recommended that TC be placed in thin layers and adequately light cured in 1 mm increments [57].

## 5. Conclusions

In conclusion, this study identified diverse gene expression and functional enriched pathways of human deciduous tooth-derived dental pulp cells treated with TC and MTA for different durations. Thirty-three significantly enriched pathways involved in TC were predicted using KEGG pathway analysis. Among the enriched pathways, neuroactive ligand–receptor interaction pathway and calcium signaling pathway may primarily contribute to the human deciduous tooth-derived dental pulp cells response after TC application. The results of this study demonstrated TC expressed genes involved two enriched KEGG pathways which may be associated with osteoclastogenesis and osteoclastic differentiation. These findings suggest that TC should be applied with caution when direct pulp capping is indicated in primary teeth.

## Figures and Tables

**Table 1:** Identified KEGG pathways involved in TC.

Entry ID	KEGG Pathway	Number of Genes	Involved Genes	*p*-Value	FDR *q*-Value^1^
hsa04080	Neuroactive ligand–receptor interaction	22	GRM5, ADORA2B, ADRA1D, CHRM2, HTR7, PTAFR, TACR1, C3AR1, CHRNA1, CHRNA3, CHRNB1, LTB4R, ADORA1, FPR1, GABRA1, GLRA2, GRM6, OPRL1, P2RY2, TRPV1, KISS1R, GALR2	1.19 × 10^−7^	2.22 × 10^−5^
hsa04020	Calcium signaling pathway	14	GRM5, ADORA2B, ADRA1D, CHRM2, HTR7, PTAFR, TACR1, PRKCB, PLCG2, CAMK2A, CAMK4, PDE1A, ATP2B2, TNNC2	2.96 × 10^−5^	2.75 × 10^−3^

^1^ FDR, false discovery rate (*q* < 0.05).

**Table 2:** Top five KEGG pathways significantly enriched in TC compared to MTA in a time-dependent manner.

Entry ID	KEGG Pathway	Number of Genes	*p*-Value	FDR *q*-Value^1^
**Upregulated in 24 h** ^2^				
hsa00350	Tyrosine metabolism	6	1.85 × 10^−4^	3.43 × 10^−2^
**Downregulated in 24 h**				
hsa04660	T cell receptor signaling pathway	17	7.8 × 10^−11^	1.45 × 10^−8^
hsa05322	Systemic lupus erythematosus	17	3.85 × 10^−9^	3.58 × 10^−7^
hsa04650	Natural killer cell mediated cytotoxicity	16	2.38 × 10^−8^	1.48 × 10^−6^
hsa04060	Cytokine–cytokine receptor interaction	21	1.6 × 10^−7^	6.71 × 10^−6^
hsa04940	Type I diabetes mellitus	9	1.8 × 10^−7^	6.71 × 10^−6^
**Upregulated in 72 h**				
hsa04080	Neuroactive ligand–receptor interaction	28	3.52 × 10^−10^	6.54 × 10^−8^
hsa05412	Arrhythmogenic right ventricular cardiomyopathy	11	2.30 × 10^−6^	2.14 × 10^−4^
hsa04062	Chemokine signaling pathway	15	9.26 × 10^−5^	5.74 × 10^−3^
hsa04060	Cytokine-cytokine receptor interaction	17	4.23 × 10^−4^	1.61 × 10^−2^
hsa05414	Dilated cardiomyopathy	9	4.34 × 10^−4^	1.61 × 10^−2^
**Downregulated in 72 h**				
hsa04010	MAPK signaling pathway	24	1.52 × 10^−7^	2.82 × 10^−5^
hsa04510	Focal adhesion	18	4.83 × 10^−6^	4.50 × 10^−4^
hsa05200	Pathways in cancer	23	1.56 × 10^−5^	9.67 × 10^−4^
hsa04514	Cell adhesion molecules (CAMs)	12	1.83 × 10^−4^	8.52 × 10^−3^
hsa04512	ECM-receptor interaction	9	3.24 × 10^−4^	1.20 × 10^−2^

^1^ FDR, false discovery rate (*q* < 0.05). ^2^ Only one KEGG pathway was significantly enriched in this category.

**Table 3:** Summary of DEGs in KEGG pathway involved in TC for 24 h and 72 h.

Genes	Description	KEGG Pathway^1^	FC^2^ Ratio (TC/MTA)		Normalized FC (log2)			
					24 h		72 h	
			24 h	72 h	MTA	TC	MTA	TC
ADORA1	Adenosine A1 receptor	1	4.749	2.766	0.795	3.043	1.046	2.514
ADORA2B	Adenosine A2b receptor	1,2	2.889	0.403	1.332	2.863	3.217	1.905
ATP2B2	ATPase plasma membrane Ca2+ transporting 2	2	0.288	9.435	1.822	0.028	0.025	3.263
CAMK4	Calcium/calmodulin dependent protein kinase IV	2	4.339	4.554	0.744	2.861	0.045	2.232
C3AR1	Complement C3a receptor 1	1	0.368	2.872	1.454	0.012	0.011	1.533
GRM5	Glutamate metabotropic receptor 5	1,2	0.374	0.489	1.437	0.017	1.033	0.000
PLCG2	Phospholipase C gamma 2	2	0.405	2.285	2.368	1.064	2.052	3.244
P2RY2	Purinergic receptor P2Y2	1	2.652	2.344	2.556	3.963	2.382	3.611

^1^ KEGG pathway; 1 = neuroactive ligand receptor interaction, 2 = calcium signaling pathway, ^2^ FC, fold change.

## Appendix A

**Table A1:** DEGs involved in neuroactive ligand–receptor interaction in TC for 24 h and 72 h.

Gene	Description	FC^2^ Ratio (TC/MTA)		Normalized FC (log_2_)			
				24 h		72 h	
		24 h	72 h	MTA	TC	MTA	TC
GRM5	Glutamate metabotropic receptor 5	0.374	0.489	1.437	0.017	1.033	0.000
ADORA2B	Adenosine A2b receptor	2.889	0.403	1.332	2.863	3.217	1.905
CHRM2	Cholinergic receptor muscarinic 2	2.066	0.139	0.000	1.047	2.843	0.000
HTR7	5-hydroxytryptamine receptor 7	0.420	3.231	4.639	3.386	2.386	4.078
PTAFR	Platelet activating factor receptor	3.093	0.244	0.000	1.629	2.038	0.000
TACR1	Tachykinin receptor 1	0.360	2.425	3.122	1.647	1.640	2.918
C3AR1	Complement C3a receptor 1	0.368	2.872	1.454	0.012	0.011	1.533
CHRNA1	Cholinergic receptor nicotinic alpha 1 subunit	0.253	0.323	4.387	2.403	6.530	4.899
CHRNA3	Cholinergic receptor nicotinic alpha 3 subunit	0.462	0.372	2.159	1.045	2.358	0.930
CHRNB1	Cholinergic receptor nicotinic beta 1 subunit	2.937	2.494	2.900	4.454	3.231	4.550
LTB4R	Leukotriene B4 receptor	0.351	0.475	5.485	3.977	5.671	4.598
ADORA1	Adenosine A1 receptor	4.749	2.766	0.795	3.043	1.046	2.514
FPR1	Formyl peptide receptor 1	0.197	11.638	4.009	1.666	1.657	5.198
GABRA1	Gamma-aminobutyric acid type A receptor alpha1 subunit	2.056	4.751	0.000	1.040	0.017	2.265
GLRA2	Glycine receptor alpha 2	4.091	0.327	0.000	2.032	1.615	0.000
GRM6	Glutamate metabotropic receptor	0.298	4.122	2.808	1.059	1.052	3.096
OPRL1	Opioid related nociception receptor 1	0.304	2.337	4.371	2.652	2.383	3.608
P2RY2	Purinergic receptor P2Y2	2.652	2.344	2.556	3.963	2.382	3.611
TRPV1	Transient receptor potential cation channel subfamily V member 1	0.311	3.241	3.346	1.661	2.384	4.081
KISS1R	KISS1 receptor	0.365	0.327	1.463	0.010	1.612	0.000
GALR2	Galanin receptor 2	0.378	0.374	1.423	0.021	2.356	0.936

^1^ KEGG pathway; 1 = neuroactive ligand–receptor interaction, 2 = calcium signaling pathway, ^2^ FC, fold change.

**Table A2:** DEGs involved in calcium signaling pathway in TC for 24 h and 72 h.

Gene	Description	FC^2^ Ratio (TC/MTA)		Normalized FC (log_2_)			
				24 h		72 h	
		24 h	72 h	MTA	TC	MTA	TC
GRM5	Glutamate metabotropic receptor 5	0.374	0.489	1.437	0.017	1.033	0.000
ADORA2B	Adenosine A2b receptor	2.889	0.403	1.332	2.863	3.217	1.905
ADRA1D	Adrenoceptor alpha	3.113	0.134	2.325	3.964	4.370	1.469
CHRM2	Cholinergic receptor muscarinic 2	2.066	0.139	0.000	1.047	2.843	0.000
HTR7	5-hydroxytryptamine receptor 7	0.420	3.231	4.639	3.386	2.386	4.078
PTAFR	Platelet activating factor receptor	3.093	0.244	0.000	1.629	2.038	0.000
TACR1	Tachykinin receptor 1	0.360	2.425	3.122	1.647	1.640	2.918
PRKCB	Protein kinase C beta	0.221	2.862	2.191	0.014	0.013	1.530
PLCG2	Phospholipase C gamma	0.405	2.285	2.368	1.064	2.052	3.244
CAMK2A	Calcium/calmodulin dependent protein kinase II alpha	0.305	6.701	2.783	1.068	1.642	4.387
CAMK4	Calcium/calmodulin dependent protein kinase IV	4.339	4.554	0.744	2.861	0.045	2.232
PDE1A	Phosphodiesterase 1A	0.455	0.387	2.786	1.649	2.856	1.485
ATP2B2	ATPase plasma membrane Ca2+ transporting 2	0.288	9.435	1.822	0.028	0.025	3.263
TNNC2	Troponin C2, fast skeletal type	0.480	3.034	3.119	2.060	1.641	3.242

^1^ KEGG pathway; 1 = neuroactive ligand–receptor interaction, 2 = calcium signaling pathway, ^2^ FC, fold change.
